# Micro and Nanoplastics Identification: Classic Methods and Innovative Detection Techniques

**DOI:** 10.3389/ftox.2021.636640

**Published:** 2021-02-26

**Authors:** Stefania Mariano, Stefano Tacconi, Marco Fidaleo, Marco Rossi, Luciana Dini

**Affiliations:** ^1^Department of Biological and Environmental Science and Technology, University of Salento, Lecce, Italy; ^2^Department of Biology and Biotechnology “Charles Darwin”, Sapienza University of Rome, Rome, Italy; ^3^Department of Basic and Applied Sciences for Engineering, Sapienza University of Rome, Rome, Italy; ^4^Research Center for Nanotechnologies Applied to Engineering, CNIS Sapienza University of Rome, Rome, Italy; ^5^National Research Council Nanotec, Lecce, Italy

**Keywords:** microplastics, nanoplastics, characterization, microscopy, spectroscopy, analytical methods, environmental matrices

## Abstract

Micro and nanoplastics are fragments with dimensions less than a millimeter invading all terrestrial and marine environments. They have become a major global environmental issue in recent decades and, indeed, recent scientific studies have highlighted the presence of these fragments all over the world even in environments that were thought to be unspoiled. Analysis of micro/nanoplastics in isolated samples from abiotic and biotic environmental matrices has become increasingly common. Hence, the need to find valid techniques to identify these micro and nano-sized particles. In this review, we discuss the current and potential identification methods used in microplastic analyses along with their advantages and limitations. We discuss the most suitable techniques currently available, from physical to chemical ones, as well as the challenges to enhance the existing methods and develop new ones. Microscopical techniques (i.e., dissect, polarized, fluorescence, scanning electron, and atomic force microscopy) are one of the most used identification methods for micro/nanoplastics, but they have the limitation to produce incomplete results in analyses of small particles. At present, the combination with chemical analysis (i.e., spectroscopy) overcome this limit together with recently introduced alternative approaches. For example, holographic imaging in microscope configuration images microplastics directly in unfiltered water, thus discriminating microplastics from diatoms and differentiates different sizes, shapes, and plastic types. The development of new analytical instruments coupled with each other or with conventional and innovative microscopy could solve the current problems in the identification of micro/nanoplastics.

## Introduction

The strong impact of environmental plastic pollution on the development, growth, and survival of several living species, including humans, has prompted the scientific community to develop new monitoring and decontamination systems. Despite the various advantages in daily use, the consumption of plastic materials increases environmental pollution due to their low biodegradability, inappropriate use, and inefficient disposal. The exposure of plastic derivatives in the environment promotes physical, chemical, and biological degradation processes leading to the accumulation of small plastic fragments both in the terrestrial and aquatic ecosystems, i.e., freshwater (Eerkes-Medrano et al., 2015), sediments (Yang et al., 2020), soil (Li et al., 2020), air (Prata, 2018), and foodstuff (Kwon et al., 2020).

In function of their size, plastic fragments can be classified in macro- and mesoplastics (> 5 mm), microplastics (MPs, <5 mm) and nanoplastics (NPs, with a range size from 1 nm to 1 mm) (Gigault et al., 2018; Yong et al., 2020). Depending on the mechanism of release: MPs can be categorized into primary and secondary MPs. Primary MPs are purposefully manufactured in that form and are released directly in form of small-sized particles in the environment by different non-biological matrixes; for example, primary MPs are abundantly released from cosmetic and body care products (i.e., microbeads in scrub gel, body and facial cleaners, cosmetics, etc.), laundering of synthetic clothes and abrasion of tires through driving. Secondary MPs derive from the abiotic and biotic degradation of large plastics after their environment exposure (Wu P. et al., 2019). Sources of secondary MPs include household usage, industrial manufacturing, debris from disposed car tires, etc. (Nizzetto et al., 2016; Wu P. et al., 2019; Yang et al., 2020). It has been estimated that secondary MPs represent 70–80% of MPs released into the environment while only 15–31% are primary MPs. In addition, several physical properties are used to classify MPs, such as density (light/heavy), flexibility (hard/soft) or shape (fragments, pellets, filaments, and granules) (Hidalgo-Ruz et al., 2012). The structure and composition of MPs are closely related to the source material and the most abundant plastic polymers of which they are composed are polyethylene (PE), polypropylene (PP), polystyrene (PS), polyvinylchloride (PVC), nylon (PA), cellulose acetate (CA), and thermoplastic polyester (PET) (Campanale et al., 2020). In the environment, MPs are easily transported and spread by wind and water currents due to their low density and size; these characteristics and their resistance to biological degradation make MPs particularly bio-accumulative and resistant to environmental decomposition. Syberg et al. (2015) showed that MPs degradation is a function of their physical and chemical properties (i.e., shape, size, porosity, surface area, morphology, and solubility) and their ability to interact with other contaminants (Syberg et al., 2015; Campanale et al., 2020). For the purposes of production, in fact, plastics are often produced with the addition of chemical additives of different nature, potentially dangerous for health. A large variety of these toxic compounds have been found in MPs, such as plasticizers, pigments, antioxidants, acid scavengers, flame retardants, light and heat stabilizers, lubricants, antistatic agents, and heath stabilizer (Hahladakis et al., 2018). In addition to these chemical additives, MPs may also have adsorbed hydrophobic or hydrophilic organic pollutants from the environment due to their high surface areas and affinity for these contaminants (Mei et al., 2020). For example, persistent organic pollutants, like aromatic hydrocarbons, polychlorinated biphenyls, and phthalates, have been identified in MPs collected from the environment (Campanale et al., 2020). In fact, MPs are found often linked to toxic chemicals acting as a vector for their transport into the environment. While MPs transport toxic chemicals into ecosystems, they are themselves, on the other hand, a cocktail of hazardous chemicals that are voluntarily added during their production as additives to increase the properties of polymers and prolong their life.

Toxic chemicals, attached through adsorption processes to MPs, can cause effects in both the biotic and abiotic environment, as they can be ingested by inhalation or contact (Bradney et al., 2019; Campanale et al., 2020). Desorption processes allow toxic compounds to be released after ingestion and thus exert potential toxicity and/or be accumulated in the food chain. Once disperse in the environment, the MPs interaction with the different species present, can influence the MPs behavior. For example, interactions between pelagic and benthic microbial communities and MPs change the characteristics of pollutant over time and define how and why cells attach themselves to plastic particles. As a result of their ingestion and transfer into food webs, the consumers' internal exposure to these environmental contaminants is affected (Rogers et al., 2020).

Regarding risk assessment, the limited data in the literature on the amounts of MPs and NPs dispersed into the environment require an assessment calculated using commercially available model particles to determine whether the size-specific toxicity paradigms established for the others engineered nanomaterials also apply to MPs/NPs (Giese et al., 2018). MPs consist of a set of materials that differ not only in the particle characteristics, such as size and shape, but also in chemical composition (including polymers, additives, etc.). So far, it is not known whether the plastic chemicals or the particle itself are the factor causing the toxicity of MPs. Indeed, the literature reports studies also showing minimal effects of MPs/NPs on a range of species such as bacteria, yeast, phyto- and protozoa, nematodes (Hanna et al., 2016, 2018; Heinlaan et al., 2020), but many other studies demonstrate different toxic effects of MPs and related contaminants in invertebrates (Foley et al., 2018), vertebrates (Miranda et al., 2019), seabirds (Duis and Coors, 2016), and mammals (Yong et al., 2020). Although no cases of death have been recorded after consumption of MPs, their ingestion reduces somatic growth rates, altered metamorphosis, lower reproductive capacity, and oxidative damage (Jeong et al., 2017; Foley et al., 2018; Leung et al., 2018; Ziajahromi et al., 2018) in invertebrate animals. In vertebrates, exposure to MPs can occur by ingestion of other exposed organisms or by direct absorption of plastic fragments from contaminated water columns or sediments. For example, a study by Veneman et al. (2017) reports systemic toxic effects in *Danio rerio* (Zebrafish) larvae induced by polystyrene MPs (Veneman et al., 2017). Similarly, the exposure of *Danio rerio* to PA, PE, PP, and PVC MPs caused intestinal alterations, including villi disruption and splitting of enterocytes, with an increased glutathione *S*-transferase (GST) activity correlated with oxidative damage (Lei et al., 2018).

The ubiquitous presence of MPs in the environment and in everyday products makes human exposure to MPs inevitable. The main entry routes of these micro-sized plastics into the human body are ingestion, inhalation, and skin exposure. The physical and morphological properties of MPs influence the entry. Indeed, the size, shape, and surface area of MPs strongly influence diffusion processes through biological barriers, adhesion to the epithelium and bioaccumulation in deep tissues (Prata et al., 2020a). Similarly, in recent *in vitro* studies, the absorption, translocation and cytotoxic effects of engineered NPs are affected by their size, charge, and shape (Elliott et al., 2017; Stock et al., 2019; Wu B. et al., 2019). The bioaccumulation potential of MPs increases with decreasing size, indicating both *in vivo* and *in vitro* the widespread risk of exposure to health (Deng et al., 2017; Schirinzi et al., 2017). Ingestion of contaminated food is considered the main route of exposure to MPs, which is related to potential effects on the gastrointestinal system, impaired epithelial permeability, localized inflammatory processes and changes in the composition of gut microbiota (Campanale et al., 2020). In addition to ingestion, inhalation, and contact with the respiratory tract represent a dangerously risky route of exposure due to the high rate of diffusion of MPs in the air flows. Aside from differences in metabolism and individual susceptibility, the response to MPs inhalation can be summarized as immediate bronchial reactions, diffuse interstitial fibrosis, inflammatory and fibrotic changes in bronchial and peribronchial tissue, and interalveolar lesions (Prata, 2018). Conversely, the diffusion through the *stratum corneum* is limited to nano-sized plastics below 100 nm, so absorption of MPs through the skin is improbable (Revel et al., 2018). Once in the bloodstream, MPs can reach peripheral tissues triggering various toxic effects, such as oxidative stress, inflammatory processes, metabolic disturbances, neurotoxic effects, etc. Accumulation of MPs in the liver and spleen has been described in mouse models after exposure by ingestion and inhalation (Jani et al., 1990; Eyles et al., 2001). Furthermore, the oral administration of polystyrene MPs induces accumulations in the gut, intestine, and kidney, with evident effects on the redox balance, alteration in energy homeostasis and neurotoxicity (Deng et al., 2017).

Monitoring of MPs in various biotic and abiotic environmental matrices is necessary to define the state of pollution, flow and risk of exposure by organisms. Monitoring studies require reliable and comparable methods. However, identifying MPs of different composition, shape, and size with a single technique is a rather difficult goal.

In general, the analysis of MPs consists of two phases: physical characterization of the displayed fragments, followed by chemical characterization thus confirming the chemical nature of the particles found. Microscopical techniques (i.e., stereo, fluorescence, atomic force, transmission, and scanning electron microscopy) are the most exploited strategies to achieve the goal. The main objective of this review is to describe the microscopical and analytic methods for the detection, characterization and identification of MPs in different environmental matrices. Among the analytical methods, chemical characterization techniques such as spectroscopy and thermal analysis will be described, although some microscopic techniques, such as TEM, SEM, and fluorescence microscopy have an analytical potential that allows to identify and determine the chemical and physical properties of many polymers. We discuss the advantages and limitations of the different microscopic and analytical techniques (reported in Table 1 together with the estimated TRL present level for each method), as well as their combination and new approaches to solve MPs/NPs identification issues.

**Table 1:** Advantages and limitations of the current methods for MPs characterization and the relative estimated TRL present range.

**Identification method**	**Advantages**	**Limitations**	**Estimated TRL present range**
Stereo microscopy	- Fast and easy - Identification of shape, size, and colors	- Not confirmative of plastic nature of the particle - No polymer composition results - Lack of data of transparent or small particles	5–7
Transmission Electron Microscopy	- Very high resolution (<0.1 nm) - Elemental analysis of particles if coupled with EDS - Analytical capabilities with EELS	- Very time expensive - Require sample preparation for particle size > 100 nm	1–2
Scanning Electron Microscopy	- Clear and high-resolution images of particles - Elemental analysis of particles if coupled with EDS - No gas into the chamber if coupled in ESEM mode - No sputtering if coupled in ESEM mode - Small detected particles in STEM mode - No treatment of sample in FE-SEM mode	- Expensive - Long time and effort for analysis - Lack of information on the type of polymer	4–6
Atomic Force Microscopy	- No radiation damage of the sample - Preserved sample surface - 3D images of the surface structure of the polymers - Best resolution obtained (0.3 nm)	- No prevention from outside factors like contaminations - Damage caused by the interaction of the tip with the sample	2–4
Fluorescence microscopy	- Easy - Detection of transparent particles - Immediate visualization of the particles	- Laser in the ultraviolet can be harmful and toxic for the sample - Chemical additives can interfere with fluorescence	4-6
Raman Spectroscopy	- Detection of small MPs (1 μm) and NPs (<1 μm) - No false positive or negative data - Non-destructive analysis of materials - Analysis of samples in solution, gas, film, surface, solids and single crystals is possible	- Expensive instrumentation - Time-consuming - Interference with pigments - Possible fragments released by adhesive polymers	5–7
FTIR spectroscopy	- Confirmation of the composition of the MPs - No false positive or negative data - Detection of small plastic particles (less of 20 μm) with μ-FTIR - Non-destructive analysis of materials	- Expensive - Wavelength radiation can be a limiting detection factor - Time consuming to analyse all the particles on a filter	5–7
Thermal analysis	- Characterization of low-solubility MPs and additives	- Destructive technique - Complex data	3–5

## Identification Methods

### Microscopy

#### Stereo- (or Dissecting) Microscopy

The stereomicroscope allows three dimensions analysis by observing the sample from two slightly different angles to obtain the two images necessary for stereoscopic vision. Thus, it is possible to observe objects mainly by means of reflected light at low magnification, typically between 8 and 50 times. The illumination system of stereoscopic microscopes comes from above, conversely to the optical microscope in which the light beam comes from below and crosses the sample. In some stereomicroscope models there is a double lighting. Differing from the optical microscope with higher magnifications, it is therefore suitable for the observation of whole fresh microorganisms, cells, fungi, and permanent slides. Although the lower magnification of the stereomicroscope may seem like a limit, it has, in fact, advantages for its very easy to use for studying objects that can be observed with the naked eye.

**Figure 1:**
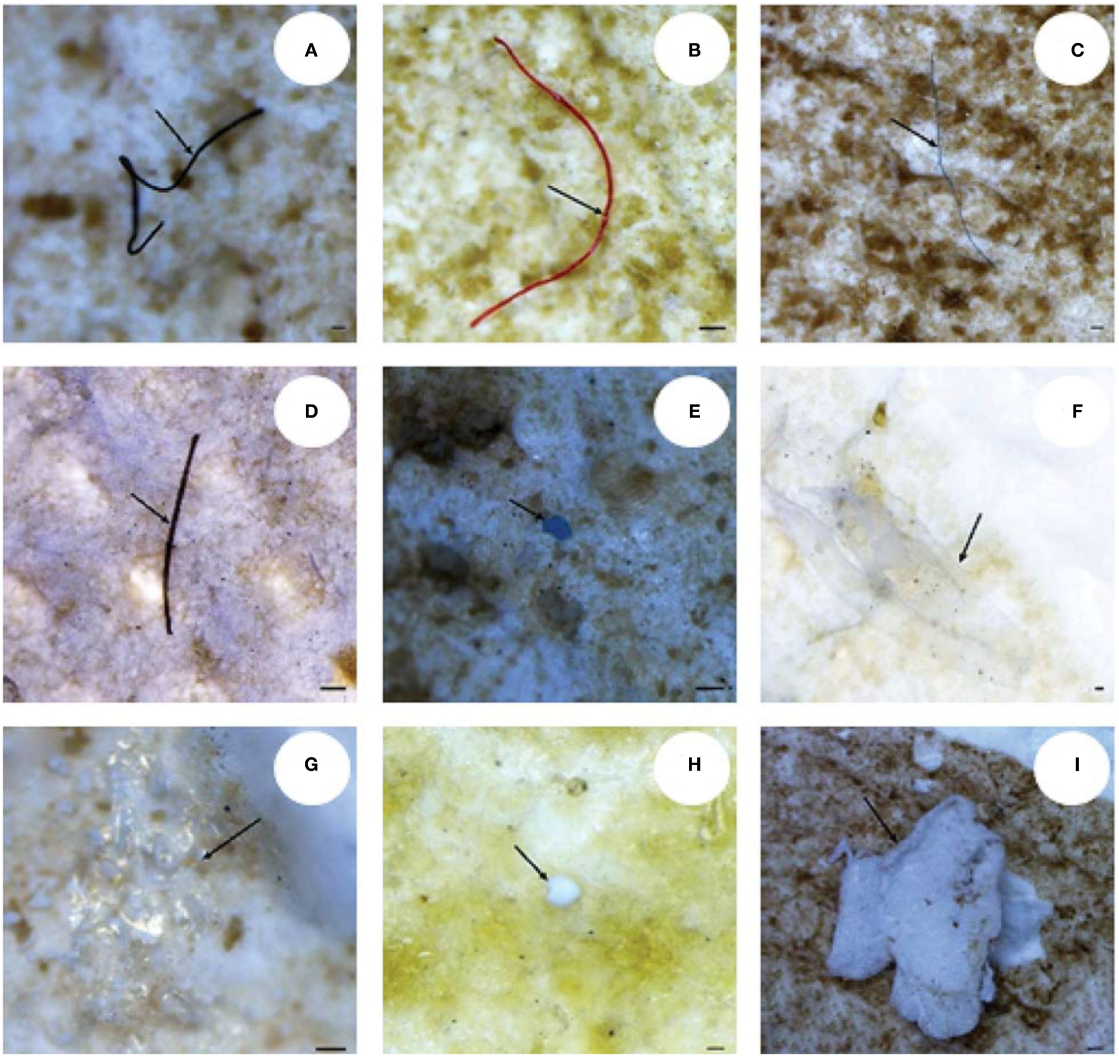
Images of different shapes of MPs in biological samples. The arrows indicate fibers **(A–D)**, fragments **(E,F)**, the film **(G)** and granules **(H,I)**. Scale bar = 100 μm. This figure is reproduced from Ding et al. (2019) with permission of Royal Society of Chemistry (RSC).

The stereomicroscope is a useful and widely used for the identification of MPs whose dimensions fall within the range of hundreds of microns. Figure 1 reports some examples of different shapes of MPs in seawater samples. Magnified microscope images provide detailed surface structure and structural information of the objects, essential for identifying the ambiguous plastic particles typology. Although many particles in the size of few microns are visible under the microscope, particles smaller than 100 μm that are transparent or have a particular shape are difficult to characterize (Song et al., 2015). Also, particularly dense sediment samples can interfere with the microscopic identification of MPs on filter paper. Also, when a sample contains material that cannot be eliminated by chemical digestion, the identification is compromised.

Several studies report that the percentage of plastic-like particles identified by stereomicroscopy, and subsequently characterized by other techniques, is about 20–70% of the total particles found, in the case of transparent particles (Eriksen et al., 2013; Song et al., 2015). Furthermore, synthetic and natural fibers (very abundant elements in water, sediment, and biota samples) are difficult to identify using stereomicroscope (Browne et al., 2010; Lusher et al., 2013). Stereomicroscope is used to identify MPs based on their physical appearance. This is a first fast-screening method that allows rapid identification of shape, size, and color of the particles which will be further characterized by other methods. Hence, the need to couple the stereomicroscopy with other techniques such as spectroscopy.

#### Fluorescence Microscopy

Unlike the optical microscope which relies on the contrast of the image given by the reflection of light on the sample, the fluorescence microscope collects fluorescent emission from the samples that are excited by a specific wavelength. The sample to be analyzed, consisting mainly of living organisms such as cells, bacteria, or tissues, contains a substance called fluorophore, which may already be present in the sample to be analyzed (it can act as fluorophore for proteins or neurotransmitters), or be introduced from the outside. This substance is hit and excited by the laser light, then, in turn, emits fluorescence light. In traditional techniques the laser generally emits in the visible (blue) and in the UV continuously, passes through a thin hole (pinhole) and focuses on a point of the sample to be observed. Since the wavelength is quite short, each fluorophore molecule that is hit by a photon is excited and emits fluorescence in the focus and the area surroundings the focus. The wavelength of the exciter laser must be ~50–200 nm shorter than the light emitted by the fluorophore because the incident photon has to supply more energy. For example, a fluorophore can absorb a wavelength of 360 nm (in the ultraviolet spectrum) and emit a wavelength of 450 nm, i.e., in the blue green light spectrum. In a Confocal laser scanning microscope, the light beam does not illuminate all the preparations but only a small part (point). Consequently, the detector does not see a complete image but only the intensity of light coming from a point of the preparation. By sliding the point along a plane or even in a volume, it is possible to reconstruct the image of the sample in sections or in three dimensions. There are many limitations to this excitation system. First, the excitation of the fluorophore not only occurs in the focus, where the laser power is concentrated, but also above and below the focal plane, in the cone that forms the laser by focusing. This generates unwanted fluorescence even in the vicinity of the focus, partly eliminated by the pinhole, with an added background noise that reduces the signal-to-noise ratio and therefore the resolution. The in-depth scan of the samples and the three-dimensional scans are particularly limited. Furthermore, the ultraviolet laser can be harmful and toxic to the sample, when analyzing organic material. The effect of the UV laser is phototoxicity and photodecay (bleaching of the sample), reducing the sample observation time.

Fluorescence microscopy has been used both for studies of biological samples (cells, bacteria, etc.) and for MPs (Ettinger and Wittmann, 2014; Prudent and Raoult, 2019). The ingestion of MPs/NPs by *T. japonicas* copepod has been examined by fluorescence microscopy (Lee et al., 2013). Three different sizes of microbeads were selected to evaluate the effect of microplastic size on the survival, development, and fecundity of *T. japonicas*.

Fluorescence microscopy is a useful strategy, particularly for white and transparent plastics, to identify MPs based on their innate ability to emit fluorescence (Noren, 2008). This strategy reduces the MPs detection failure and can lower the size limit of the detected MPs when combined with imaging. The detection of MPs in different matrices can also be based on the quantitation of fluorescent spheres with microscopy techniques (Batel et al., 2018; Dawson et al., 2018; Catarino et al., 2019, Schür et al., 2019). So far, however, the investigation of biological membrane crossing evidence by MPs has been inconclusive, as labeled NPs solutions may have residual free dye that peels off in an altered medium (i.e., pH, salts, or biological affinity) and the intensity of total fluorescence in cells after exposure can be mistakenly attributed to the presence of nanoparticles, when in fact it is caused by free fluorophore (Tenuta et al., 2011; Andreozzi et al., 2013). Another limitation in the visualization of MPs is given by the chemical additives used in the synthesis process which can also influence the fluorescent properties (Piruska et al., 2005). For example, additives may exhibit fluorescent properties and interfere with microscopic fluorescence measurements (Lee et al., 2020). Therefore, it is necessary to eliminate these impurities as much as possible with adequate pre-treatment (Elert et al., 2017). Surface rinsing with acids or oxidants (e.g., hydrogen peroxide) and enzymatic digestion is usually used (Löder et al., 2017). These pre-treatments can only remove surface impurities or contaminants, but they do not reduce potential inferences from the chemicals contained within MPs.

A novel technique used for identification of MPs utilizes a fluorescent dye, i.e., Nile Red, to label plastic fragments, in particular when analyzing tissues or organisms (Cole et al., 2013). Details will be described in the paragraph “new approaches and new identification strategies”.

#### Transmission Electron Microscopy

The electron microscope has a very high-resolving power that allows the observation of samples of infinitesimal sizes thanks to the wave properties the electrons, emitted by a very thin filament of thermoionic material (W or LaB_6_) or by a field emitter source, for the top-level TEMs. Then, the electrons pass into the magnetic capacitor through a hole located in the anode (the capacitor has the purpose of regulating the intensity of the convergence of the electron beam). Transmission electron microscopy (TEM) is the most used technique in characterizing nanomaterials in electron microscopy, providing chemical information and images of nanomaterials at a spatial resolution equal to the level of atomic dimensions (Dini et al., 2015). The electron beam through which incident light is transmitted *via* a thin foil specimen is transformed into elastically or inelastically scattered electrons when the electron beam interacts with the specimen. Detection methods are in an early stage of development and no NPs have actually been detected in soft matrices by TEM, so far. TEM is not effective to visualize NPs because of their amorphous structure; in fact, NPs and MPs are not electrondense and heavy-metal stains are required. Polymers are generally composed of elements that show poor contrast in TEM because their elastic interactions with electrons are weak. As such, the use of heavy element stains remains a popular method that enables the visualization of the microstructure of organic specimens in TEM (Sawyer et al., 2008). However, the stains themselves have been shown to change the chemical structure of the polymers. The electrons are inelastically scattered as a result of various physical phenomena: phonon excitation, plasmon excitation (collective oscillations of valence electrons), and the ionization of core shell electrons. These inelastically scattered electrons are able to be analyzed spectroscopically using a technique called electron energy loss spectroscopy (EELS) (Egerton, 2011). The usual configuration of EELS is in the form of an attachment to a TEM (which provides the electron source). This makes EELS a powerful tool to probe the chemical structure of polymers with high spatial resolution as well as to study the dielectric properties of the film. Furthermore, TEM can be fluorescence-integrated that provides fluorescent information displayed as a color overlay on TEM images useful to provide some form of chemical characterization (Sims and Hardin, 2007).

Thus, TEM is not included among the techniques used in the characterization of MPs. Indeed, TEM is much used in the study of the effects of MPs on model systems. For example, Sun et al. (2018) investigated the toxic effects of polystyrene nano- and MPs on the marine bacterium *Halomonas alkaliphila* by determining growth inhibition, chemical composition, inorganic nitrogen conversion efficiencies and reactive oxygen species (ROS) generation. An increased extracellular polymeric substances as possible bacterial protective mechanisms were observed by using TEM. In another study, in order to evaluate the possible effect of MPs (PP, PE, PET, and PVC) on microalgae, as the inhibition growth and the cell structure variation, TEM characterization was performed (Song et al., 2020). The need to characterize MPs based on their size, surface characteristics, thickness, and other physicochemical properties pushes toward the use of other microscopic techniques such as SEM, fluorescence microscopy and AFM, as described below.

#### Scanning Electron Microscopy

**Figure 2:**
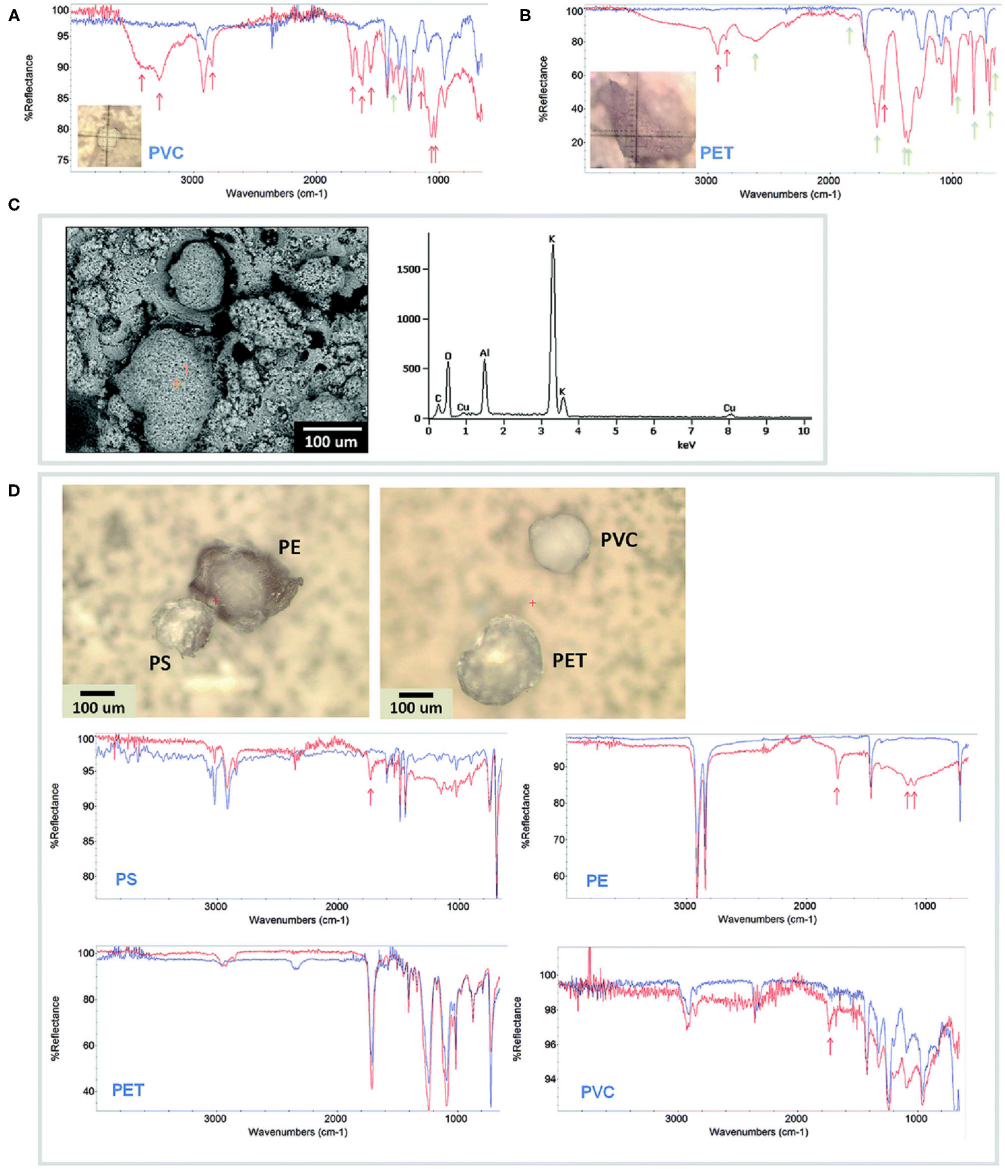
Microplastic particles extracted from laboratory medaka GI tracts. For all FTIR spectra, extracted MPs (red) are compared to the original reference MPs (blue). **(A)** 150 μm PVC and **(B)** 300 μm PET particles prepared in 10% KOH, showing strong FTIR peaks for proteins and fats (red arrows) and potassium salts (green arrows). **(C)** SEM/EDS of KOH-treated microplastic showing redeposited particulate material and strong potassium peak. **(D)** 150 μm PS, 250 μm PE, 250 μm PET, and 150 μm PVC extracted with ultrapure H_2_O and PUE, exhibiting reduced FTIR protein and fat peaks and no salt peaks. This figure is reproduced from Wagner et al. (2017) with permission of Royal Society of Chemistry (RSC).

Scanning electron microscopy (SEM) is a microscopic technique able to provide information about the morphological surface structure of MPs, generating high-resolution images of the surface state. Furthermore, it can provide data about the chemical composition of the samples, since it can be equipped with detectors for EDS (Energy Dispersive X-ray-Spectroscopy) (Figure 2C). The primary electrons penetrate the solid specimen, and different (both elastic and inelastic) scattering processes are generated, and the related different signals are collected by different detector systems to make an image (Bogner et al., 2007). In particular, the secondary electrons provide a detailed image that helps to understand the morphology of objects, while the back-scattered electrons passing through the specimen produce an increasing intensity that provides information about the topography and contrast of the material based on the atomic number (Z). Samples with elements composed of a higher atomic number (Z) produce more back-scattered electrons than lower Z elements. This phenomenon can be used to distinguish differences in the composition of samples (Reimer, 1993). EDS is not a separate technique from SEM, but an additional detector which allows the observer to get qualitative and quantitative information about the elemental analysis of the sample. It consists of an electron beam generated by an electron microscope cathode. When primary electron beam hits the surface of the sample many interactions are generated, in particular X-ray. These return data about the elements and their spatial distribution which characterize the sample.

The SEM-EDS is often considered an expensive technique. < Instead, as for other microscopic analysis, the technique requires little time and low costs. Indeed, for the characterization of MPs, it is enough to use SEM in environmental SEM mode, which avoids the use of gas-like nitrogen into the SEM chamber. In addition, coverage by sputtering gold or carbon on the sample surface can also be avoided, although it is useful for obtaining high resolution images. However, in this case, the sample would no longer be usable for other analyses, e.g., EDS or FT-IR. Several studies utilized conventional SEM to visualize MPs in different matrices: sewage sludge (Mahon et al., 2017), mussels (Li et al., 2018a), sediments (Wang and Wang, 2018; Shruti et al., 2019; Neto et al., 2020), and sand (Tiwari et al., 2019).

Naji et al. (2019) adopted, as an alternative to SEM, the FE-SEM which works on a low voltage and allows to obtain high quality and high magnification images of MPs samples without special treatments on the sample before observation. It is a quick and easy technique because it is no necessary to cover the sample with metal or carbon, but the fragments of MPs are directly placed on the carbon tape on the aluminum stub. In this case, however, the low voltage used cannot be exploited to make an EDS analysis as well. When investigating light elements, it is possible to adopt moderate energy for the analysis of non-conductive particles, avoiding adding additional signals in the EDS spectrum resulting from coverage. Otherwise, it is necessary to use higher energy which however can cause a charge effect of the sample.

At last, SEM is a versatile instrument allowing also the NPs to characterized since it can be equipped with a STEM holder to analyse sample on a grid. Small particles can be visualized without high energy and voltage typical of classical TEM (Mitrano et al., 2019).

#### Atomic Force Microscopy

The need to perform measurements on non-conductive materials without resorting to surface metallization led to the use of atomic force microscopy (AFM), which, although like the other previous microscopies in some general characteristics, is based on a completely different operating principle. In AFM, a small tip of very rigid conductive material is fixed to the end of a rod or micro-lever (cantilever) that presses the tip on the sample during the process of measure. The cantilever flows on the surface of a sample which moves along the three Cartesian axes through a movement induced by a piezoelectric mechanism. A system of control allows the tip to be kept in conditions of “constant force” (to acquire information on the interaction strength between the sample surface and the tip) or of “constant height” (to acquire information on variations in height of the sample). The oscillations of the cantilever are detected by an optical system which also records very slight movements of the bar that supports the tip. The actual image of the sample is reconstructed by processing the information relating to (i) relative movement mode between sample and tip and (ii) punctual results on the sample-tip distance (Haggerty and Lenhoff, 1993). According to the interaction modes of the tip with the sample surface, AFMs can be used in:

- Contact mode: the tip is in physical contact with the surface to be analyzed and responsible for the bending of the lever are the Van der Waals repulsive forces and the electrostatic interactions, with an average value of 10^−9^ N. The lever must have a small elastic constant, thus avoiding excessive pressure to the sample and altering its surface. The height of the lever can be kept constant or can be adjusted by the feedback loop to avoid damaging the tip. This method of analysis is used for hard samples, which are not damaged by contact with the tip. The most critical phase of the analysis is the engagement, that is the approach of the probe to the surface: an error in this process can lead to the tip breaking or to the damage of the sample surface.
- Non-contact mode: the tip is kept at a distance of a few nanometers from the sample and is sent into resonance in order to obtain a continuous vibration. Variations in oscillation frequency due to Van der Waals interaction forces depend on the distance of the tip from the sample and are used to reconstruct the surface image. This type of analysis is used with materials that are too soft, which could be damaged by contact with the tip.
- Tapping mode: this is the method that allows maximum resolutions but requires levers with high elastic constants. The lever is vibrated at frequencies close to that of resonance and is kept at a distance such that the tip makes transient contact with the sample surface when the amplitude of the oscillation is maximum. The force applied by the tip is absorbed without damage by the sample in most cases unless dealing with particularly soft materials. Changes in the amplitude of the oscillation are recorded to obtain the image.

In AFM, different types of forces that are established between sample and tip can be used to produce images. In the “no contact mode,” the images are produced by van der Waals forces, or by electro-static, magnetic and capillary forces. In the “contact mode,” the ion repulsion forces prevail. Furthermore, the friction force acting between the tip and the surface is particularly important for a complete and detailed analysis of the sample. In addition to be an indicator of the properties of the sample, friction or “lateral force” or “lateral deflection” provides information about the interaction between the tip and the surface.

AFM has been used extensively in the characterization of several nanoscale samples, from engineered nanoparticles (Fu and Zhang, 2018) to soil particles (Cheng et al., 2009), polymeric membranes (Fu and Zhang, 2017), and other nanostructures (Nguyen et al., 2017). In comparison with SEM analysis, AFM allows additional characterizations to be performed including stiffness (Zhang et al., 2012), hydrophobicity (Fu and Zhang, 2018), conductivity (Trotsenko et al., 2016), or magnetization (Middea et al., 2015).

As all microscopic techniques, the AFM also presents advantages and limitations. Advantages: (i) AFM produces images with a high resolution of a few nm; (ii) AFM preserves the sample surface as it involves simple sample preparation; (iii) AFM can be used to investigate the surface of non-conducting polymers, as in the case of MPs; (iv) AFM provides direct 3D images of the surface structure of the polymers. Using lateral force, phase contrast modes, or nanomechanical imaging, it permits the discrimination between the type of materials on the surface of polymer blends; (v) AFM is useful for the analysis of nanocomposite materials; (vi) AFM avoids radiation damage of the sample as a combination for SEM and TEM; (vii) the best resolution obtained is 0.3 nm, which is better than SEM. Recently, the technique has been exploited to study the process of degradation of MPs in marine environmental, in presence of adherent bacteria on the surface (Dussud et al., 2018). In another study, Luo et al. (2020) successfully prepared a nano-TiO2-coated polypropylene MPs, and explored the nanoscale infrared, thermal, and mechanical properties of MPs before and after photoaging combing AFM with analytical technique. However, AFM presents some limitation. First, AFM cannot prevent outside factors like contaminations; also, the tip can produce artifacts derived, for example, from the damage caused by the interaction of the tip with the sample (Jagtap and Ambre, 2006). Moreover, the contact could release fragments, in case of adhesive polymers, to the tip and could produce an incorrect image of the sample (Ukraintsev et al., 2012).

Thus, AFM is a promising method to characterize MPs or NPs, but a combination with other techniques is needed. In the following paragraphs, the coupling with other analytical techniques will be better described.

### Analytical Methods

#### FT-IR Spectroscopy

Infrared spectroscopy or IR spectroscopy is an absorption spectroscopic technique normally used in the field of material characterization for the study of chemical bonds. When an infrared photon is absorbed by a molecule, it passes from its fundamental vibrational state to an excited vibrational state.

In a typical infrared spectrum on the abscissa we find the wavenumber of the incident photon and on the ordinate the transmittance. The vibrations can be of two types: stretching of the chemical bond (stretching) and deformation of the bond angle (bending). Fourier transform IR or FTIR spectroscopy is performed using an interferometer, which allows the scanning of all the frequencies present in the IR radiation generated by the source. Scanning is possible thanks to a moving mirror which, by moving, introduces a difference in the optical path, which causes constructive or destructive interference with the ray reflected from a fixed mirror. In this way, an interferogram is obtained which shows the representation of intensity in the time domain. By applying the Fourier transform, the interferogram is transformed into a spectrum with peaks corresponding to specific chemical bonds or molecular vibration (Smith, 2011). FTIR involves four techniques: transmission (Turner and Holmes, 2011), reflectance (Guo et al., 2017), true specular reflectance/reflection-adsorption (Rodenko et al., 2018) and attenuated total reflection (Song et al., 2014). Among the main advantages of FT-IR, which guarantees higher performance, the high availability of energy results in a much better signal/noise ratio than traditional IR spectroscopy. Furthermore, the analysis times are significantly reduced.

Together with Raman spectroscopy, FTIR is much used to characterize MPs (Lefebvre et al., 2019; Schwabl et al., 2019; Corami et al., 2020). Figures 2A,B,D show the FTIR spectra of some MPs extracted from Medaka *(Oryzias latipes)* gastrointestinal tracts. In every study, MPs samples are excited giving specific detected vibrations which allow a spectrum with a fingerprint range to be obtained. This spectrum describes the nature of the material, which can be identified by comparing it with the known reference spectra. Larger particles (>500 nm) can be analyzed by using ATR-FTIR, but small particles require the micro-FTIR (μ-FTIR) that permit to get a simultaneous visualization, mapping, and collection of spectra. It can be performed both in ATR and reflectance mode. Jung et al. (2018), for example, provided a definitive validation of ATR FTIR to identify ingested plastic polymer types in sea turtle. ATR permits to obtain high-quality spectra but needs infrared-transparent substrate. The reflectance mode is used for thick samples, but irregular surfaces of particles can interfere with analysis because of refractive error (Harrison et al., 2012). Therefore, only samples with particular properties can be analyzed, otherwise, reftractive errors prevent you from having a correct signal. Furthermore, the lateral resolution is limited to a diffraction range and sample with a dimension of < 20 μm are not detectable (Löder et al., 2015). μ-FTIR have been widely used in MPs works to find and characterize them within the sediment (Harrison et al., 2012; Peng et al., 2017), marine organisms (Zhang et al., 2020), surface water (Wu P. et al., 2019), food (Li et al., 2018b). The technique allows to collect IR signals at a high spatial resolution, also it is useful for the characterization of complex samples. For example, Zhang et al. (2020) performed a μ-FTIR analysis on microplastic pollution in surface sediments from 28 stations in Sishili Bay, identifying eight polymer types including rayon, PE, PP, PA, PET, PS, PMMA, and PU.

As already described above, AFM is used to scan the surfaces of materials and then generate an image of their height; however, the technique cannot easily identify the molecular composition of the observed materials. Researchers developed a combination of AFM and IR spectroscopy, called the AFM-IR technique. AFM-IR is a widely used method for the acquisition of IR absorption spectra and absorption images with a spatial resolution of 50–100 nm (Dazzi and Prater, 2017). It is achieved by coupling AFM equipment with a pulse tunable IR source, which has a pulse length of 10 ns and covers a wide range of mid-infrared regions. The AFM-IR microscope uses a micro-lever, connected at one end to the support, while a sharp tip is connected to the other end; the measurement of the imperceptible movements of the introduced sample is carried out using an infrared laser beam. The absorption of light by the sample generates a slight expansion, with a consequent deflection of the micro-lever, which therefore produces an infrared signal. Thermal expansion of the sample due to the absorbing of pulsed light by a sample produces heat and generates an impulse on AFM cantilever, causing the cantilever to oscillate, which is called a “ring-down” (Dazzi et al., 2015). In this study, AFM-IR technique in different modes was used to explore the nanoscale infrared, thermal and mechanical properties of TiO_2_-pigmented MPs before and after aging. AFM topographical images showed that the surface of unaged MPs was relatively smooth, but the aged MPs had a rough surface with more granular domains. Combined AFM-IR technique and photothermal infrared spectroscopy are used to locate, image, and chemically identify the beads in the mussel siphons (Merzel et al., 2019). Some works have successfully exploited the technique by investigating samples that were known to be contaminated with plastic particles. Indeed, the characterization of unknown samples can highlight some limits of the technique as it is tough and time-consuming to scan and find nano-sized particles in an unknown sample.

#### Raman Spectroscopy

Raman spectroscopy is a technique based on the interaction between radiation and material. This technique exploits a laser radiation that interacts with the vibrational motions of the molecules and causes the re-emission of light at wavelengths that are characteristic of those specific atomic groups. A Raman spectrum is therefore generated by the inelastic scattering between the photons of an incident radiation and the molecules that constitute the sample (Ribeiro-Claro et al., 2017). By irradiating the sample with a monochromatic light beam at frequency ν0, a part of the radiation is elastically diffused at the same initial frequency ν0, i.e., with photons of the same energy (a phenomenon defined as Rayleigh scattering). The spectrum of the scattered radiation will also present a series of lines with a higher or a lower frequency than the Rayleigh line, due to inelastic diffusion (Kauffmann et al., 2019).

Raman spectroscopy is another useful chemical analytical technique for identification of MPs in different environmental matrices: Van Cauwenberghe et al. (2013) analyzed plastic particles sized in the micrometer range in deep-sea sediments collected at four locations representing different deep-sea habitats ranging in depth from 1100 to 5000 m; Cole et al. (2013) identified that 13 zooplankton taxa had the capacity to ingest 1.7–30.6 μm polystyrene beads, with uptake varying by taxa, life-stage and bead-size; Murray and Cowie (2011) found balls of tightly tangled plastic threads were found in the intestines of the Norway lobster, *Nephrops norvegicus* and Raman spectroscopy indicated that some of the microfilaments identified by the intestinal contents could be derived from fishing waste; Imhof et al. (2013) collected sediment samples from two beaches at Lake Garda for the identification of MPs. Detection and quantification were performed using Raman micro-spectroscopy which allows the analysis of particles down to the μm-range. Conventional Raman usually detects MPs larger than 10 μm, while micro-Raman spectroscopy (μ-Raman spectroscopy) permits to analyse MPs up to 1 μm. It is an integration between the Raman spectroscope and an optical microscope that makes it possible to visually select the specific area of the sample to be analyzed. This allows it to be used in many and varied scientific fields, with applications above all in the environmental, forensic, materials sciences, biology and medicine, geology, pharmaceuticals, and restoration of works of art fields. In the last years, it has been used at the university research level for the identification of MPs in bottled water in order to analyse the release of MPs from the packaging (Di et al., 2019; Tong et al., 2020). Other applications of μ-Raman spectroscopy are reported in Table 2, regarding the identification of MPs in different environmental matrices like sediment, organisms, freshwater, drink, cosmetics, etc.

**Table 2:** Application of μ-Raman spectroscopy in MPs characterization.

**Origin/matrix/reference**	**MPs type**	**MPs size**	**Results**
Northwestern Pacific/seawater (Pan et al., 2019)	PE, PP, NYLON	0.5 and 1.0 mm	μ-Raman spectroscopic analysis results indicate that the major compositions of MPs are PE (57.8%), PP (36.0%) and nylon (3.4%) and the origin of the MPs at these stations are the same, namely, the nearby land in Philippines, Taiwan, and China Mainland
Caspian Sea/sediment (Mehdinia et al., 2020)	PS, PE	250–500 μm	μ-Raman spectroscopy detected only PS and PE in studied samples. In general, the polymer types indicated lower diversity in comparison with those reported in such areas in the world.
East Dongting Lake/sediment (Yin et al., 2020)	PET, PP, PE PS, PA, PVC, PMMA, CL	0.05–5 mm	Eight types of MPs with different polymer compositions were identified by μ-Raman spectroscopy. The study found that the abundance of MPs in the urban area sediment of Dongting Lake is lower than that of the rural area.
Germany/soil (Paul et al., 2019)	PE, PP, PS, PET	< 125 μm	MPs of the materials PE, PP, PS, PET, and PVC can be detected in soils at levels of about 1 mass% after minimal conditioning, e.g., sieving and drying of the material
Italy/white wine (Prata et al., 2020b)	PE	38–475 μm	μ-Raman spectroscopy was used for the first time in complex beverages in the identification of MPs particles in white wines, allowing identification of at least one synthetic particle for each bottle, except in two cases.
Malaysia/fish meals (Karbalaei et al., 2020)	PE, PP	855.82 μm ± 1082.90SD	Chemical composition of extracted MP-like particles was confirmed using μ-Raman spectroscopy. Out of 336 extracted particles, 64.3% were plastic polymers, 25% pigment particles, 4.2% non-plastic items, and 6.5% were unidentified. Fragments were the dominant form of MPs (78.2%) followed by filaments (13.4%) and films (8.4%).

Raman spectroscopy presents many advantages with respect to FTIR spectroscopy. It allows non-destructive analysis of materials, in any state of aggregation, with generally reduced sample preparation using lasers of different power able to affect the material, generating a Raman spectrum characteristic of the analyzed material. Also, the thickness of samples is not important in the measurement. Analysis of samples in solution, gas, film, surface, solids, and single crystals is possible. Analysis can be performed at various temperatures. The low-temperature spectra (10 K) allow: (i) to minimize any damage caused to the sample by local heating induced by the laser; (ii) can be compared with studies obtained with other methods that provide good results at low temperatures. Among the disadvantages, fluorescence can be a very serious problem if combined with Raman spectroscopy. In fact, the Raman and the fluorescence are intimately connected since in both phenomena the photon emitted comes from excitation in the absorption band and the quantum yield of the fluorescence is often of an order of magnitude higher than the intensity of the Raman diffusion. However, some fluorescence interference can be overcome by applying an algorithm or more efficient detectors (Araujo et al., 2018).

#### Thermal Analysis

Apart from different IR absorption or Raman scattering properties, plastic polymers also differ in their thermal stability. The MPs identification through thermo-analytical techniques exploits changes in the physical and chemical properties of polymers (Majewsky et al., 2016). The methodology is based on the identification of the polymer according to its degradation products. The development of thermal methods is pivotal for the characterization of low-solubility MPs and additives that cannot be easily dissolved, extracted, or hydrolysed. The thermal analysis includes techniques like differential scanning calorimetry (DSC), thermogravimetry (TGA), pyrolysis-gas chromatography-mass spectrometry (py-GC-MS), and combinations of these methods.

DSC is a thermal analysis technique that can be used to measure the temperature and the heat flux associated with the transitions that occur in a sample, the melting enthalpies, the glass transitions and crystallization kinetics of polymeric materials. The basic principle of this technique is to obtain information about the material by heating or cooling it in a controlled manner. In particular, the DSC is based on the measurement of the difference in heat flow between the sample under examination and a reference sample, while both are bound to a variable temperature defined by a pre-established program. DSC is a useful technique for the study of polymeric materials, but it requires reference materials. Indeed, it is mostly used for the identification of primary MPs like PE microbeads which have known characteristics (Castañeda et al., 2014). However, the use of DSC for the analysis of plastics such as polycarbonate (PC), polystyrene (PS), polymethyl methacrylate (PMMA), and acrylonitrile butadiene (ABS), among others, has limitations as these plastics melt over a wide range of temperatures. Furthermore, quantification of the mass of MPs in environmental samples has also been reported (Majewsky et al., 2016; Shim et al., 2016). Among other limitations, DSC results in a lack of specificity when characterizing a mixture of MPs with closely spaced melting points (Majewsky et al., 2016) and the overlap of melting peaks.

Thermogravimetry (TGA) is another classical technique that allows the quantitative thermal analysis of a sample by measuring the weight lost from the sample at a certain temperature, however, without identifying the nature of the components. From this analysis, it is possible to obtain graphs (the mass as function of the temperature) thermogravimetric details. It is a thermal analysis technique widely used in the case of polymeric materials. It is known how the thermal degradation mechanism of polymers can be significantly influenced by the experimental conditions in which the heating is performed. Therefore, the reproducibility of polymer thermogravimetry data requires as detailed control as possible of the operating conditions of the experiment, such as the size and the shape of the sample, the rate of heating, the type of atmosphere in which the sample is heated (Peñalver et al., 2020). In TGA a loss of mass is measured, whereas the degradation of polymeric materials begins frequently with enthalpy changes. Enthalpy changes cannot be detected in TGA and are obtainable by DSC measurements. Therefore, a combination of two methods is suggested in MPs analysis (Golebiewski and Galeski, 2007). Many MPs can be identified, but for other polymers detection is impossible due to their overlapping phase transition signals (Majewsky et al., 2016).

One of the analytical techniques that has been successfully applied in the analysis of plastic materials is Py-GC-MS. It is ideal for simultaneously identifying and quantifying the most abundant MPs in complex samples. The semiquantitative calculation for each type of plastic present, particularly for trace concentrations (ppb or lower), represents the specific identification of the polymer in environmental and animal samples and the external calibration curve. Research software and libraries dedicate to polymers and additives speed up identification. The analytical method is independent of mechanical preselection or particle appearance, however proper sample preparation is essential and needs to be evaluated according to the nature and origin of the sample (Peñalver et al., 2020). The sample, with a mass up to 350 mg, is thermally degraded in an inert atmosphere and the resulting fragments of the polymer structure can be separated by GC and characterized by MS.

The information obtained by the technique derives from the type of chemical product derived from pyrolysis. Each polymer is characterized by its own degradation products and ions that are exploited for identification (Chen et al., 2019; Yang et al., 2019). It has advantages and limitations. First of all, the method does not require any pre-treatment of the sample, it also allows the characterization of polymers and additives present in the sample and reduces time and cost compared to other solvent extraction techniques used in the analysis of marine MPs particles (Fries et al., 2013). One of the main limitations of the characterization of Py-GC-MS for MPs is the lack of particle size information. Therefore, in some studies this technique has been coupled with others such as microscopy and FTIR Primpke et al. (2020), for example, compared the results of hyperspectral FTIR imaging analysis and Py-GC/MS analysis performed on a set of environmental samples that differ in complexity and degree of microplastic contamination. With this combination, they harmonized data sets that are either mass or particle number related.

The thermal analysis gives an alternative method to spectroscopy for some polymer's identification. However, it is a destructive technique that prevent to analyse MPs with other following methods. For this reason, it is disadvantageous to use these analytical methods for MPs identification, but they could be useful for the first screening of bulk samples to be later analyzed by spectroscopy.

## New Approaches and New Identification Strategies

As we have described above, the detection of MPs usually requires a microscope. However, the method still has limitations, due to the possibility to perform characterization and identification (Lenz et al., 2015; Song et al., 2015). Therefore, it is expected to be coupled with analytical techniques. An FT-IR or Raman spectroscope equipped with a microscope has generally been used for the chemical identification of polymers at the microscale, including qualitative confirmation of polymer types (Peng et al., 2017; Pan et al., 2019; Mehdinia et al., 2020; Prata et al., 2020b; Zhang et al., 2020). They also allow the identification of MPs with dimensions of tens of microns, but it is often necessary to repeat the analysis several times to obtain reliable spectra of very small plastic particles. Analytical methods require expensive instruments and a very long analysis time, especially in the case where the number of particles to be investigated is high. This applies not only to environmental samples with complex matrices but also to the quantification of controlled laboratory experimental samples. Therefore, it is necessary to develop new alternative approaches that allow a quick and simple identification of MPs both to be monitored in the field and to be investigated in the laboratory, evaluating their toxicity, accumulation, aging, etc.

**Figure 3:**
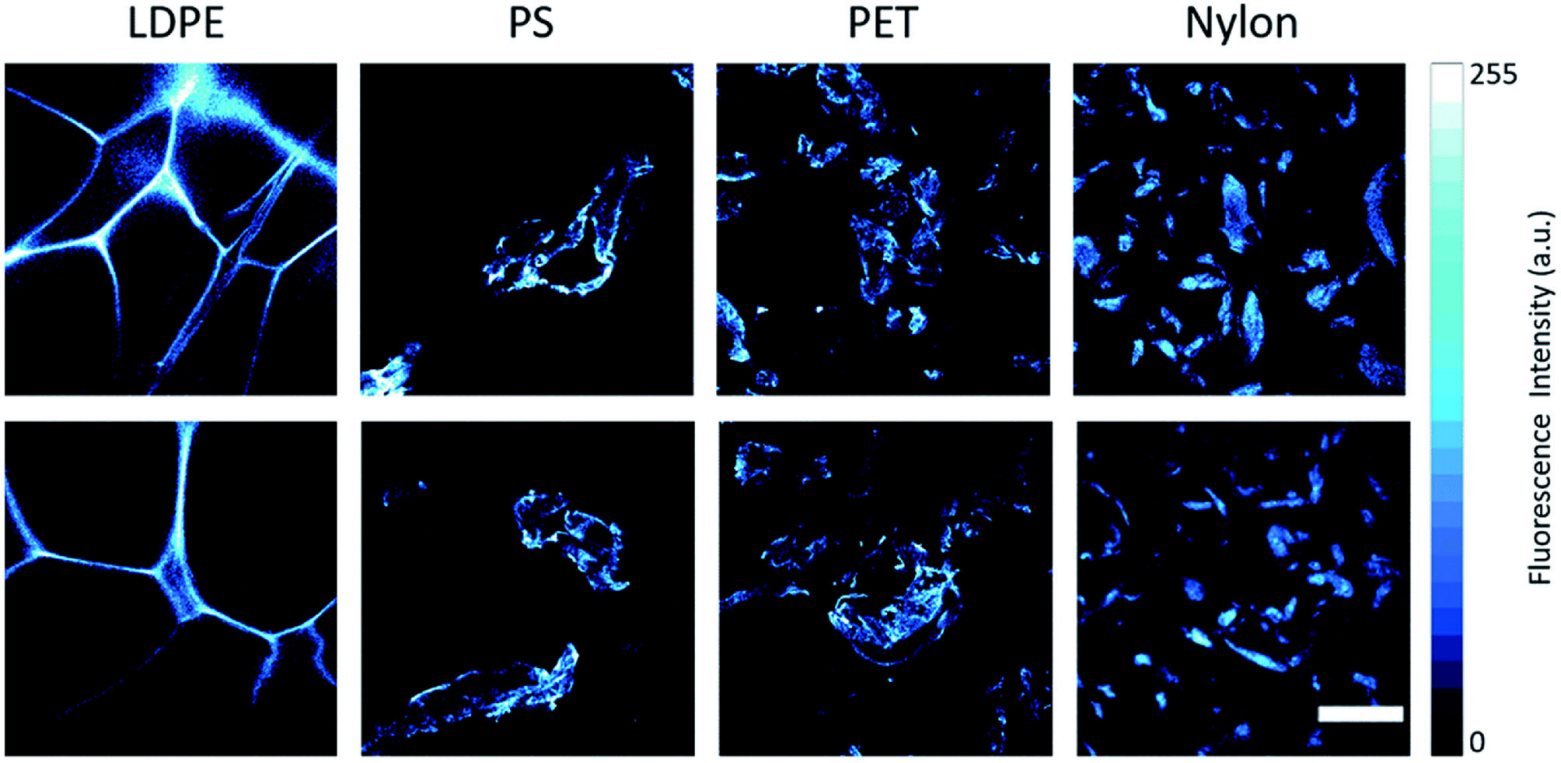
Representative confocal fluorescence microscopy images of MPs dispersed in water and stained with Nile Red. From left to right: low-density polyethylene (LDPE), polystyrene (PS), polyethylene terephthalate (PET), and polyamide (nylon). Fluorescence emission signals are acquired in the range of 520–720 nm at λex = 500 nm. The scale bar is 200 μm. This figure is reproduced from Sancataldo et al. (2020) with permission of Royal Society of Chemistry (RSC).

A staining technique could provide a new alternative or complementary method to address these problems. “Nile Red” (9-diethylamino-5-benzo[a]phenoxazinone; NR) is a dye which, by binding preferentially to polymeric materials rather than organic ones, is able to allow rapid detection and quantification of MPs thanks to its absorption, selectivity and fluorescent properties (Figure 3). The dye adsorbs onto plastic surfaces and renders them fluorescent when irradiated with blue light (Maes et al., 2017). Although contested by some, this method remains today one of the most advanced among those available, sufficient by itself to identify a particle of a polymeric nature without the need for further spectroscopic analyses (thus reducing the time required to analyse an environmental sample).

It is the solution proposed by a team of researchers from the University of Warwick, in the United Kingdom, which has shown, after numerous tests on different types of plastic polymers, how this substance, NR, is selectively and targeted when it comes into contact with certain chemicals (Erni-Cassola et al., 2017). One of the limitations of the method is the co-staining of natural organic material. Thus, the first step of the scientists was to verify, during the experimentation, that the dye did not also identify products similar to MPs such as fatty substances or small fragments of wood, for this reason, the team of researchers decided to wash the analyzed particles with nitric acid, a substance effective in the “digestion” of all types of biogenic material. The next step was the sampling of sand and water on the coast of the city of Plymounth and the comparison between the measurements obtained with traditional methods and the new fluorescent color. The result highlighted a greater amounts of MPs <1 mm (0.04 inch) compared to that obtained with traditional methods. In particular, from the analyses, it emerged that the type of plastic most present was PP, a polymer that is used, among many uses, for different types of packaging and for banknotes. Many studies adopted this technique for MPs identification in biological tissues of *Hydra attenuate* (Gagné et al., 2019); in water bottles (Mason et al., 2018); in earthworm *Eisenia fetida* in soil (Wang et al., 2019); in terrestrial invertebrates biomass (Maxwell et al., 2020).

NR staining is an useful technique to quickly identify MPs and, at the same time, it is a step for sample preparation before carrying out the spectroscopic analysis. If a fluorescent filter is applied on FT-IR microscope, the combination of the two methods can reduce the non-identified MPs in the samples, as well as the time to check every plastic-like particle (Shim et al., 2016; Iannilli et al., 2019; Gaston et al., 2020).

Recently, another study conducted by researchers from the CNR Institute of Applied Sciences and Intelligent Systems (Isasi-Cnr) has identified a new method, called digital holography (DH), capable of distinguishing MPs from microplankton or microalgae within marine samples (Bianco et al., 2020). Using artificial intelligence and a holographic sensor, the method proposed allows information to be acquired from the analyzed elements thanks to the use of a holographic microscope. This provides a wide and unprecedented range of highly distinctive parameters that characterize MPs (Mandracchia et al., 2017; Paturzo et al., 2018). The holographic microscope is a digital microscope that is based on the classic principle of holography: storage of visual information with the use of lasers whose holographic registration is carried out through a plate. The difference is that the digital microscope projects the hologram through a sensor and not on a plate. In addition, three-dimensional imaging requires three laser sources in the visible range to obtain as many images, thus receiving an increase in spatial resolution. In this way, the information acquired makes it possible to “train” an artificial intelligence system, which will thus be able to distinguish the polluting material from other natural materials, whose dimensions and shapes are very similar to those of MPs. DH allows the problem of detecting MPs present in a water sample to overcome. It is an optical technique for 3D imaging that is quantitative and completely label-free. The use of a digital camera without mechanical scanning allows the recognition of transparent objects at different depths, investigating the presence of MPs in large volumes of water (Merola et al., 2018). Thanks to this technique, it is possible to carry out the detection, counting and quantitative measurement of the physical dimensions of the MPs in the entire volume. The combination of digital holography and artificial intelligence makes possible to recognize tens of thousands of objects belonging to different classes with an accuracy > 99%. Moreover, the new digital holography method, provides objective recognition of a statistically significant number of samples, up to hundreds of thousands of objects per hour, with microscopes that can be made in portable configurations for *in situ* water quality analysis.

This new and alternative technique has only recently been proposed, so there are no other groups in the literature that have adopted this method in the detection of MPs.

## Conclusions and Future Perspectives

In recent decades, plastic has been produced and used by humans with increasing frequency, so much so that, to date, this material has become the largest polluting anthropogenic debris in the environment. However, there is a lack of information about the presence of MPs in different environmental matrices such as their effect on human health and rapid monitoring.

Currently, there is no unique method of identification and characterization that can be efficient for each different case examined here. In this review, we have reported the different techniques most used to detect and characterize MPs in environmental samples, highlighting their advantages and limitations. The combination of microscopic techniques with analytical methods could overcome some problems highlighted. Recent studies introduced other approaches that improve this research area as those described at the end of this review. It is good to remember that there are still no sufficiently valid methods to identify MPs quickly and without a doubt. It is essential to develop new methods that are reliable and practical and that can indicate the future direction of development of methods and tools for identifying MPs.

## Author Contributions

SM and ST write the manuscript. MF performed bibliographic research. MR contributed to constructive revising of the manuscript. LD designed, write, and critically revised the manuscript. All authors contributed to the article and approved the submitted version.

## Conflict of Interest

The authors declare that the research was conducted in the absence of any commercial or financial relationships that could be construed as a potential conflict of interest.

## References

[B1] Andreozzi P. Martinelli C. Carney R. P. Carney T. M. Stellacci F. (2013). Erythrocyte incubation as a method for free-dye presence determination in fluorescently labeled nanoparticles. Mol. Pharm. 10, 875–882. 10.1021/mp300530c 23190092

[B2] Araujo C. F. Nolasco M. M. Ribeiro A. Ribeiro-Claro P. (2018). Identification of microplastics using Raman spectroscopy: latest developments and future prospects. Water Res. 142, 426–440. 10.1016/j.watres.2018.05.060 29909221

[B3] Batel A. Borchert F. Reinwald H. Erdinger L. Braunbeck T. (2018). Microplastic accumulation patterns and transfer of benzo[a]pyrene to adult zebrafish (*Danio rerio*) gills and zebrafish embryos. Environ. Pollut. 235, 918–930. 10.1016/j.envpol.2018.01.028 29751397

[B4] Bianco V. Memmolo P. Carcagn I. P. Merola F. Paturzo M. Distante C.. (2020). Microplastic identification via holographic imaging and machine learning. Adv. Intell. Syst. 2:1900153. 10.1002/aisy.201900153

[B5] Bogner A. Jouneau P. H. Thollet G. Basset D. Gauthier C. (2007). A history of scanning electron microscopy developments: towards “wet-STEM” imaging. Micron 38, 390–401. 10.1016/j.micron.2006.06.008 16990007

[B6] Bradney L. Wijesekara H. Palansooriya K. N. Obadamudalige N. Bolan N. S. Ok Y. S.. (2019). Particulate plastics as a vector for toxic trace-element uptake by aquatic and terrestrial organisms and human health risk. Environ. Int. 131:104937. 10.1016/j.envint.2019.104937 31284110

[B7] Browne M. A. Galloway T. S. Thompson R. C. (2010). Spatial patterns of plastic debris along Estuarine shorelines. Environ. Sci. Technol. 44, 3404–3409. 10.1021/es903784e 20377170

[B8] Campanale C. Massarelli C. Savino I. Locaputo V. Uricchio V. F. (2020). Detailed review study on potential effects of MPs and additives of concern on human health. Int. J. Environ. Res. Public Health 17:1212. 10.3390/ijerph17041212 PMC7068600 32069998

[B9] Castañeda R. A. Avlijas S. Simard M. A. Ricciardi A. (2014). Microplastic pollution in St. Lawrence river sediments. Can. J. Fish. Aquat. Sci. 71, 1767–1771. 10.1139/cjfas-2014-028125590075

[B10] Catarino A. I. Frutos A. Henry T. B. (2019). Use of fluorescent-labelled nanoplastics (NPs) to demonstrate NP absorption is inconclusive without adequate controls. Sci. Total Environ. 670, 915–920. 10.1016/j.scitotenv.2019.03.194 30921723

[B11] Chen M. F. Liu Y. H. Lin J. H. Liu C. P. (2019). Characterization of a novel silicon containing hybrid polymer by thermal curing, pyrolysis behavior, and fluorescence analysis. J. Appl. Polym. Sci. 136:6. 10.1002/app.47403

[B12] Cheng S. Y. Bryant R. Doerr S. H. Wright C. J. Williams P. R. (2009). Investigation of surface properties of soil particles and model materials with contrasting hydrophobicity using atomic force microscopy. Environ. Sci. Technol. 43, 6500–6506. 10.1021/es900158y 19764208

[B13] Cole M. Lindeque P. Fileman E. Halsband C. Goodhead R. Moger J.. (2013). Microplastic ingestion by zooplankton. Environ. Sci. Technol. 47, 6646–6655. 10.1021/es400663f 23692270

[B14] Corami F. Rosso B. Bravo B. Gambaro A. Barbante C. (2020). A novel method for purification, quantitative analysis and characterization of microplastic fibers using micro-FTIR. Chemosphere 238:124564. 10.1016/j.chemosphere.2019.124564 31472348

[B15] Dawson A. Huston W. Kawaguchi S. King C. Cropp R. Wild S.. (2018). Uptake and depuration kinetics influence microplastic bioaccumulation and toxicity in antarctic Krill (*Euphausia superba*). *Environ. Sci. Technol*. 52, 3195–3201. 10.1021/acs.est.7b05759 29397707

[B16] Dazzi A. Prater C. B. (2017). AFM-IR: technology and applications in nanoscale infrared spectroscopy and chemical imaging. Chem. Rev. 117, 5146–5173. 10.1021/acs.chemrev.6b00448 27958707

[B17] Dazzi A. Saunier J. Kjoller K. Yagoubi N. (2015). Resonance enhanced AFM-IR: a new powerful way to characterize blooming on polymers used in medical devices. Int. J. Pharm. 484, 109–114. 10.1016/j.ijpharm.2015.02.046 25703904

[B18] Deng Y. Zhnag Y. Lemos B. Ren H. (2017). Tissue accumulation of MPs in mice and biomarker responses suggest widespread health risks of exposure. Sci. Rep. 7:46687. 10.1038/srep46687 28436478 PMC5402289

[B19] Di M. Liu X. Wang W. Wang J. (2019). Manuscript prepared for submission to environmental toxicology and pharmacology pollution in drinking water source areas: MPs in the Danjiangkou Reservoir, China. Environ. Toxicol. Pharmacol. 65, 82–89. 10.1016/j.etap.2018.12.009 30580204

[B20] Ding J. Li J. Sun C. Jiang F. Ju P. Qu L.. (2019). Detection of microplastics in local marine organisms using a multi-technology system. Anal. Methods 11, 78–87. 10.1039/C8AY01974F

[B21] Dini L. Panzarini E. Mariano S. Passeri D. Reggente M. Rossi M.. (2015). Microscopies at the nanoscale for nano-scale drug delivery systems. Curr. Drug Targets 16, 1512–1530. 10.2174/1389450116666150531160851 26028043

[B22] Duis K. Coors A. (2016). MPs in the aquatic and terrestrial environment: sources (with a specific focus on personal care products), fate and effects. Environ. Sci. Eur. 28:2. 10.1186/s12302-015-0069-y 27752437 PMC5044952

[B23] Dussud C. Meistertzheim A. L. Conan P. Pujo-Pay M. George M. Fabre P.. (2018). Evidence of niche partitioning among bacteria living on plastics, organic particles and surrounding seawaters. Environ. Pollut. 236, 807–816. 10.1016/j.envpol.2017.12.027 29459335

[B24] Eerkes-Medrano D. Thompson R. C. Aldridge D. C. (2015). MPs in freshwater systems: a review of the emerging threats, identification of knowledge gaps and prioritisation of research needs. Water Res. 75, 63–82. 10.1016/j.watres.2015.02.012 25746963

[B25] Egerton R. F. (2011). Electron Energy-Loss Spectroscopy in the Electron Microscope. Boston, MA: Springer Science and Business Media. 10.1007/978-1-4419-9583-4

[B26] Elert A. M. Becker R. Duemichen E. Eisentraut P. Falkenhagen J. Sturm H.. (2017). Comparison of different methods for MP detection: what can we learn from them, and why asking the right question before measurements matters? Environ. Pollut. 231, 1256–1264. 10.1016/j.envpol.2017.08.074 28941715

[B27] Elliott J. T. Rösslein M. Song N. W. Toman B. Kinsner-Ovaskainen A. Maniratanachote R.. (2017). Toward achieving harmonization in a nano-cytotoxicity assay measurement through an interlaboratory comparison study. ALTEX 34, 201–218. 10.14573/altex.1605021 27684074

[B28] Eriksen M. Mason S. Wilson S. Box C. Zellers A. Edwards W.. (2013). Microplastic pollution in the surface waters of the Laurentian Great Lakes. Mar. Pollut. Bull. 77, 177–182. 10.1016/j.marpolbul.2013.10.007 24449922

[B29] Erni-Cassola G. Gibson M. I. Thompson R. C. Christie-Oleza J. A. (2017). Lost, but found with Nile red: a novel method for detecting and quantifying small MPs (1 mm to 20 μm) in environmental samples. Environ. Sci. Technol. 51, 13641–13648. 10.1021/acs.est.7b04512 29112813

[B30] Ettinger A. Wittmann T. (2014). Fluorescence live cell imaging. Methods Cell. Biol. 123, 77–94. 10.1016/B978-0-12-420138-5.00005-7 24974023 PMC4198327

[B31] Eyles J. E. Bramwell V. W. Williamson E. D. Alpar H. O. (2001). Microsphere translocation and immunopotentiation in systemic tissues following intranasal administration. Vaccine 19, 4732–4742. 10.1016/S0264-410X(01)00220-1 11535324

[B32] Foley C. J. Feiner Z. S. Malinich T. D. Höök T. O. (2018). A meta-analysis of the effects of exposure to MPs on fish and aquatic invertebrates. Sci. Total Environ. 631–632, 550–559. 10.1016/j.scitotenv.2018.03.046 29529442

[B33] Fries E. Dekiff J. H. Willmeyer J. Nuelle M.-T. Ebert M. Remy D. (2013). Identification of polymer types and additives in marine microplastic particles using pyrolysis-GC/MS and scanning electron microscopy. Environ. Sci. Proc. Improv. 15, 1949–1956. 10.1039/c3em00214d 24056666

[B34] Fu W. Zhang W. (2017). Hybrid AFM for nanoscale physicochemical characterization: recent development and emerging applications. Small 13:1603525. 10.1002/smll.201603525 28121376

[B35] Fu W. Zhang W. (2018). Measurement of the surface hydrophobicity of engineered nanoparticles using an atomic force microscope. PCCP 20, 24434–24443. 10.1039/C8CP04676J 30221292

[B36] Gagné F. Auclair J. Quinn B. (2019). Detection of polystyrene nanoplastics in biological samples based on the solvatochromic properties of Nile red: application in *Hydra attenuata* exposed to nanoplastics. Environ. Sci. Pollut. Res. Int. 26, 33524–33531. 10.1007/s11356-019-06501-3 31578681

[B37] Gaston E. Woo M. Steele C. Sukumaran S. Anderson S. (2020). MPs differ between indoor and outdoor air masses: insights from multiple microscopy methodologies. Appl. Spectrosc. 74, 1079–1098. 10.1177/0003702820920652 32233850

[B38] Giese B. Klaessig F. Park B. Kaegi R. Steinfeldt M. Wigger H.. (2018). Risks, release and concentrations of engineered nanomaterial in the environment. Sci. Rep. 8:1565. 10.1038/s41598-018-19275-4 29371617 PMC5785520

[B39] Gigault J. Baudrimont M. Pascal P. Gauffre F. Phi T. El Hadri H.. (2018). Current opinion: what is a nanoplastic? Environ. Pollut. 235, 1030–1034. 10.1016/j.envpol.2018.01.024 29370948

[B40] Golebiewski J. Galeski A. (2007). Thermal stability of nanoclay polypropylene composites by simultaneous DSC and TGA. Compos. Sci. Technol. 67, 3442–3447. 10.1016/j.compscitech.2007.03.007

[B41] Guo C. Luo X. Zhou X. Shi B. Wang J. Zhao J.. (2017). Quantitative analysis of binary polymorphs mixtures of fusidic acid by diffuse reflectance FTIR spectroscopy, diffuse reflectance FT-NIR spectroscopy, Raman spectroscopy and multivariate calibration. J. Pharm. Biomed. Anal. 140, 130–136. 10.1016/j.jpba.2017.02.053 28359962

[B42] Haggerty L. Lenhoff A. M. (1993). STM and AFM in biotechnology. Biotechnol. Prog. 9, 1–11. 10.1021/bp00019a001 7763408

[B43] Hahladakis J. N. Velisa C. A. Weberb R. Iacovidoua E. Purnella P. (2018). An overview of chemical additives present in plastics: migration, release, fate and environmental impact during their use, disposal and recycling. J. Hazard Mater. 344, 179–199. 10.1016/j.jhazmat.2017.10.014 29035713

[B44] Hanna S. K. Cooksey G. A. Dong S. Nelson B. C. Mao L. Elliott J. T.. (2016). Feasibility of using a standardized Caenorhabditis elegans toxicity test to assess nanomaterial toxicity. Environ. Sci. Nano 3, 1080–1089. 10.1039/C6EN00105J PMC11561884 39544221

[B45] Hanna S. K. Montoro Bustos A. R. Peterson A. W. Reipa V. Scanlan L. D. Hosbas Coskun S.. (2018). Agglomeration of *Escherichia coli* with positively charged nanoparticles can lead to artifacts in a standard caenorhabditis elegans toxicity assay. Environ. Sci. Technol. 52, 5968–5978. 10.1021/acs.est.7b06099 29672024 PMC6081640

[B46] Harrison J. P. Ojeda J. J. Romero-González M. E. (2012). The applicability of reflectance micro-Fourier-transform infrared spectroscopy for the detection of synthetic MPs in marine sediments. Sci. Total Environ. 416, 455–463. 10.1016/j.scitotenv.2011.11.078 22221871

[B47] Heinlaan M. Kasemets K. Aruoja V. Blinova I. Bondarenko O. Lukjanova A.. (2020). Hazard evaluation of polystyrene nanoplastic with nine bioassays did not show particle-specific acute toxicity. Sci. Total Environ. 707:136073. 10.1016/j.scitotenv.2019.136073 31869615

[B48] Hidalgo-Ruz V. Gutow L. Thompson R. C. Thiel M. (2012). MPs in the marine environment: a review of the methods used for identification and quantification. Environ. Sci. Technol. 46, 3060–3075. 10.1021/es2031505 22321064

[B49] Iannilli V. Pasquali V. Setini A. Corami F. (2019). First evidence of MPs ingestion in benthic amphipods from Svalbard. Environ. Res. 179:108811. 10.1016/j.envres.2019.108811 31622894

[B50] Imhof H. K. Ivleva N. P. Schmid J. Niessner R. Laforsch C. (2013). Contamination of beach sediments of a subalpine lake with microplastic particles. Curr. Biol. 23, R867–R868. 10.1016/j.cub.2013.09.001 24112978

[B51] Jagtap R. N. Ambre A. H. (2006). Overview literature on atomic force microscopy (AFM): basics and its important applications for polymer characterization. Indian J. Eng. Mater. Sci. 13:368.

[B52] Jani P. Halbert G. W. Langridge J. Florence A. T. (1990). Nanoparticle uptake by the rat gastrointestinal mucosa: quantitation and particle size dependency. J. Pharm. Pharmacol. 42, 821–826. 10.1111/j.2042-7158.1990.tb07033.x 1983142

[B53] Jeong C. B. Kang H. M. Lee M. C. Kim D. H. Han J. Hwang D. S.. (2017). Adverse effects of MPs and oxidative stress-induced MAPK/Nrf2 pathway-mediated defense mechanisms in the marine copepod *Paracyclopina nana*. Sci. Rep. 7:41323. 10.1038/srep41323 28117374 PMC5259799

[B54] Jung M. R. Horgen F. D. Orski S. V. Rodriguez C.V Beers K. L.. (2018). Validation of ATR FT-IR to identify polymers of plastic marine debris, including those ingested by marine organisms. Mar. Pollut. Bull. 127, 704–716. 10.1016/j.marpolbul.2017.12.061 29475714 PMC13077791

[B55] Karbalaei S. Golieskardi A. Watt D. U. Boiret M. Hanachi P. Walker T. R.. (2020). Analysis and inorganic composition of MPs in commercial Malaysian fish meals. Mar. Pollut. Bull. 150:110687. 10.1016/j.marpolbul.2019.110687 31699500

[B56] Kauffmann T. H. Kokanyan N. Fontana M. (2019). Use of stokes and anti-stokes Raman scattering for new applications. J. Raman Spectrosc. 5, 418–424. 10.1002/jrs.5523

[B57] Kwon J. H. Kim J. W. Pham T. D. Tarafdar A. Hong S. Chun S. H.. (2020). MPs in food: a review on analytical methods and challenges. Int. J. Environ. Res. Public Health 7:E6710. 10.3390/ijerph17186710 PMC7559051 32942613

[B58] Lee K. W. Shim W. J. Kwon O. Y. Kang J.-H. (2013). Size-dependent effects of micro polystyrene particles in the marine copepod *Tigriopus japonicus*. Environ. Sci. Technol. 47, 11278–11283. 10.1021/es401932b 23988225

[B59] Lee Y. K. Murphy K. R. Hur J. (2020). Fluorescence signatures of dissolved organic matter leached from MPs: polymers and additives. Environ. Sci. Technol. 54, 11905–11914. 10.1021/acs.est.0c00942 32852946

[B60] Lefebvre C. Saraux C. Heitz O. Nowaczyk A. Bonnet D. (2019). MPs FTIR characterisation and distribution in the water column and digestive tracts of small pelagic fish in the Gulf of Lions. Mar. Pollut. Bull. 142, 510–519. 10.1016/j.marpolbul.2019.03.025 31232331

[B61] Lei L. Wu S. Lu S. Liu M. Song Y. Fu Z.. (2018). Microplastic particles cause intestinal damage and other adverse effects in zebrafish *Danio rerio* and nematode *Caenorhabditis elegans*. Sci. Tot. Environ. 619–620, 1–8. 10.1016/j.scitotenv.2017.11.103 29136530

[B62] Lenz R. Enders K. Stedmon C. A. Mackenzie D. Nielsen T. G. (2015). A critical assessment of visual identification of marine microplastic using Raman spectroscopy for analysis improvement. Mar. Pollut. Bull. 100, 82–91. 10.1016/j.marpolbul.2015.09.026 26455785

[B63] Leung J. Chan K. Y. K. (2018). MPs reduced posterior segment regeneration rate of the polychaete *Perinereis aibuhitensis*. Mar. Pollut. Bull. 129, 782–786. 10.1016/j.marpolbul.2017.10.072 29100634

[B64] Li J. Green C. Reynolds A. Shi H. Rotchell J. M. (2018a). MPs in mussels sampled from coastal waters and supermarkets in the United Kingdom. Environ. Pollut. 241, 35–44. 10.1016/j.envpol.2018.05.038 29793106

[B65] Li J. Liu H. Chen J. (2018b). MPs in freshwater systems: a review on occurrence, environmental effects, and methods for MPs detection. Water Res. 137, 362–374. 10.1016/j.watres.2017.12.056 29580559

[B66] Li J. Song Y. Cai Y. (2020). Focus topics on MPs in soil: analytical methods, occurrence, transport, and ecological risks. Environ. Pollut. 257:113570. 10.1016/j.envpol.2019.113570 31767234

[B67] Löder M. G. J. Imhof H. K. Ladehoff M. Löschel L. A. Lorenz C. Mintenig S.. (2017). Enzymatic purification of MPs in environmental samples. Environ. Sci. Technol. 51, 14283–14292. 10.1021/acs.est.7b03055 29110472

[B68] Löder M. G. J. Kuczera M. Mintenig S. Lorenz C. Gerdts G. (2015). Focal plane array detector-based micro-Fourier-transform infrared imaging for the analysis of MPs in environmental samples. Environ. Chem. 12:563e581. 10.1071/EN14205

[B69] Luo H. Xiang Y. Li Y. Zhao Y. Pan X. (2020). Photocatalytic aging process of nano-TiO2 coated polypropylene microplastics: combining atomic force microscopy and infrared spectroscopy (AFM-IR) for nanoscale chemical characterization. J. Hazard Mater. 404:124159. 10.1016/j.jhazmat.2020.124159 33080556

[B70] Lusher A. L. McHugh M. Thompson R. C. (2013). Occurrence of MPs in the gastrointestinal tract of pelagic and demersal fish from the English Channel. Mar. Pollut. Bull. 67, 94–99. 10.1016/j.marpolbul.2012.11.028 23273934

[B71] Maes T. Jessop R. Wellner N. Haupt K. Mayes A. G. (2017). A rapid-screening approach to detect and quantify MPs based on fluorescent tagging with Nile red. Sci. Rep. 7:44501. 10.1038/srep44501 28300146 PMC5353725

[B72] Mahon A. M. O'Connell B. Healy M. G. O'Connor I. Officer R. Nash R.. (2017). MPs in sewage sludge: effects of treatment. Environ. Sci. Technol. 51, 810–818. 10.1021/acs.est.6b04048 27936648

[B73] Majewsky M. Bitter H. Eiche E. Horn H. (2016). Determination of microplastic polyethylene (PE) and polypropylene (PP) in environmental samples using thermal analysis (TGA-DSC). Sci. Total Environ. 568, 507–511. 10.1016/j.scitotenv.2016.06.017 27333470

[B74] Mandracchia B. Bianco V. Wang Z. Mugnano M. Bramanti A. Paturzo M.. (2017). Holographic microscope slide in a spatio-temporal imaging modality for reliable 3D cell counting. Lab. Chip. 17, 2831–2838. 10.1039/C7LC00414A 28722051

[B75] Mason S. A. Welch V. G. Neratko J. (2018). Synthetic polymer contamination in bottled water. Front. Chem. 6:407. 10.3389/fchem.2018.00407 30255015 PMC6141690

[B76] Maxwell S. H Melinda K. F Matthew G. (2020). Counterstaining to separate nile red-stained microplastic particles from terrestrial invertebrate biomass. Environ. Sci. Technol. 54, 5580–5588. 10.1021/acs.est.0c00711 32298090

[B77] Mehdinia A. Dehbandi R. Hamzehpour A. Rahnama R. (2020). Identification of MPs in the sediments of southern coasts of the Caspian Sea, north of Iran. Environ. Pollut. 258:113738. 10.1016/j.envpol.2019.113738 31838395

[B78] Mei W. Chen G. Bao J. Song M. Li Y. Luo C. (2020). Interactions between MPs and organic compounds in aquatic environments: a mini review. Sci. Total Environ. 736:139472. 10.1016/j.scitotenv.2020.139472 32473454

[B79] Merola F. Memmolo P. Miccio L. Mugnano M. Ferraro P. (2018). Phase contrast tomography at lab on chip scale by digital holography. Methods 136, 108–115. 10.1016/j.ymeth.2018.01.003 29341925

[B80] Merzel R. L. Purser L. Soucy T. L. Olszewski M. Colón-Bernal I. Duhaime M.. (2019). Uptake and retention of nanoplastics in Quagga Mussels. Global Challenges 4:1800104. 10.1002/gch2.201800104 32685193 PMC7268195

[B81] Middea A. Spinelli L. S. Souza Junior F. G. Neumann R. Gomes O. d. F. M. Fernandes T. L. A. P. (2015). Synthesis and characterization ofmagnetic palygorskite nanoparticles and their application on methylene blue remotion from water. Appl. Surf. Sci. 346, 232–239. 10.1016/j.apsusc.2015.03.080

[B82] Miranda T. Vieira L. R. Guilhermino L. (2019). Neurotoxicity, behavior, and lethal effects of cadmium, MPs, and their mixtures on pomatoschistus microps juveniles from two wild populations exposed under laboratory conditions-implications to environmental and human risk assessment. Int. J. Environ. Res. Public Health 16:2857. 10.3390/ijerph16162857 31405089 PMC6720622

[B83] Mitrano D. M. Beltzung A. Frehland S. Schmiedgruber M. Cingolani A. Schmidt F. (2019). Synthesis of metal-doped nanoplastics and their utility to investigate fate and behaviour in complex environmental systems. Nat. Nanotechnol. 14, 362–368. 10.1038/s41565-018-0360-3 30718833 PMC6451641

[B84] Murray F. Cowie P. R. (2011). Plastic contamination in the decapod crustacean *Nephrops norvegicus* (Linnaeus, 1758). *Mar. Pollut. Bull*. 62, 1207–1217. 10.1016/j.marpolbul.2011.03.032 21497854

[B85] Naji A. Nuri M. Amiri P. Niyogi S. (2019). Small microplastic particles (S-MPPs) in sediments of mangrove ecosystem on the northern coast of the Persian Gulf. Mar. Pollut. Bull. 146, 305–311. 10.1016/j.marpolbul.2019.06.033 31426160

[B86] Neto J. Rodrigues F. L. Ortega I. Rodrigues L. Lacerda A. Coletto J. L.. (2020). Ingestion of plastic debris by commercially important marine fish in southeast-south Brazil. Environ. Pollut. 267:115508. 10.1016/j.envpol.2020.115508 32916433

[B87] Nguyen T. Petersen E. J. Pellegrin B. Gorham J. M. Lam T. Zhao M.. (2017). Impact of UV irradiation on multiwall carbon nanotubes in nanocomposites: formation of entangled surface layer and mechanisms of release resistance. Carbon 116, 191–200. 10.1016/j.carbon.2017.01.097 28603293 PMC5460675

[B88] Nizzetto L. Bussin G. Futter M. N. Butterfield D. Paul G. (2016). Whitehead a theoretical assessment of microplastic transport in river catchments and their retention by soils and river sediments. Environ. Sci. Process. Impact 18, 1050–1059. 10.1039/C6EM00206D 27255969

[B89] Noren F. (2008). Small Plastic Particles in Coastal Swedish Waters. Technical Report. KIMO.

[B90] Pan Z. Guo H. Chen H. Wang S. Sun X. Zou Q.. (2019). MPs in the Northwestern Pacific: abundance, distribution, and characteristics. Sci. Total. Environ. 650, 1913–1922. 10.1016/j.scitotenv.2018.09.244 30286357

[B91] Paturzo M. Pagliarulo V. Bianco V. Memmolo P. Miccio L. Merola F.. (2018). Digital holography, a metrological tool for quantitative analysis: trends and future applications. Opt. Laser. Eng. 104, 32–47 10.1016/j.optlaseng.2017.11.013

[B92] Paul A. Wander L. Becker R. Goedecke C. Braun U. (2019). High-throughput NIR spectroscopic (NIRS) detection of MPs in soil. Environ. Sci. Pollut. Res. Int. 26, 7364–7374. 10.1007/s11356-018-2180-2 29754299

[B93] Peñalver R. Arroyo-Manzanares N. López-García I. Hernández-Córdoba M. (2020). An overview of MPs characterization by thermal analysis. Chemosphere 242:125170. 10.1016/j.chemosphere.2019.125170 31675574

[B94] Peng G. Zhu B. Yang D. Su L. Shi H. Li D. (2017). MPs in sediments of the Changjiang Estuary, China. Environ. Pollut. 225, 283–290. 10.1016/j.envpol.2016.12.064 28408187

[B95] Piruska A. Nikcevic I. Lee S. H. Ahn C. Heineman W. R. Limbach P. A.. (2005). The autofluorescence of plastic materials and chips measured under laser irradiation. Lab. Chip. 5, 1348–1354. 10.1039/b508288a 16286964

[B96] Prata J. C. (2018). Airborne MPs: consequences to human health? Environ. Pollut. 234, 115–126. 10.1016/j.envpol.2017.11.043 29172041

[B97] Prata J. C. da Costa J. P. Lopes I. Duarte A. C. Rocha-Santos T. (2020a). Environmental exposure to MPs: an overview on possible human health effects. Sci. Total Environ. 702:134455. 10.1016/j.scitotenv.2019.134455 31733547

[B98] Prata J. C. Paço A. Reis V. da Costa J. P. Fernandes A. da Costa F. M.. (2020b). Identification of MPs in white wines capped with polyethylene stoppers using micro-Raman spectroscopy. Food Chem. 331:127323. 10.1016/j.foodchem.2020.127323 32554310

[B99] Primpke S. Fischer M. Lorenz C. Gerdts G. Scholz-Böttcher B. M. (2020). Comparison of pyrolysis gas chromatography/mass spectrometry and hyperspectral FTIR imaging spectroscopy for the analysis of MPs. Anal Bioanal. Chem. 12, 8283–8298. 10.1007/s00216-020-02979-w PMC7680748 33104827

[B100] Prudent E. Raoult D. (2019). Fluorescence *in situ* hybridization, a complementary molecular tool for the clinical diagnosis of infectious diseases by intracellular and fastidious bacteria. FEMS Microbiol. Rev. 43, 88–107. 10.1093/femsre/fuy040 30418568

[B101] Reimer L. (1993). Image Formation in Low-Voltage Scanning Electron Microscopy. Bellingham, WA: SPIE Optical Engineering Press. 10.1117/3.2265074

[B102] Revel M. Châtel A. Mouneyrac C. (2018). Micro(nano)plastics: a threat to human health? Curr. Opin. Environ. Sci. Health 1, 17–23. 10.1016/j.coesh.2017.10.003

[B103] Ribeiro-Claro P. Nolasco M. M. Araújo C. (2017). Characterization of MPs by Raman spectroscopy. Compr. Anal. Chem. 75, 119–151. 10.1016/bs.coac.2016.10.001

[B104] Rodenko O. Tidemand-Lichtenberg P. Pedersen C. (2018). Low repetition rate 343 nm passively Q-switched solid-state laser for time-resolved fluorescence spectroscopy. Opt. Expr. 26, 20614–20621. 10.1364/OE.26.020614 30119370

[B105] Rogers K. L. Carreres-Calabuig J. A. Gorokhova E. Posth N. R. (2020). Micro-by-micro interactions: how microorganisms influence the fate of marine microplastics. LandO Lett. 5, 18–36. 10.1002/lol2.10136

[B106] Sancataldo G. Avellone G. Vetri V. (2020). Nile Red lifetime reveals microplastic identity. Environ. Sci. Process. Impacts 22, 2266–2275. 10.1039/D0EM00348D 33064112

[B107] Sawyer L. Grubb D. T. Meyers G. F. (2008). Polymer Microscopy. New York, NY: Springer Science and Business Media.

[B108] Schirinzi G. F. Pérez-Pomeda I. Sanchís J. Rossini C. Farré M. Barceló D. (2017). Cytotoxic effects of commonly used nanomaterials and MPs on cerebral and epithelial human cells. Environ. Res. 159, 579–587. 10.1016/j.envres.2017.08.043 28898803

[B109] Schür C. Rist S. Baun A. Mayer P. Hartmann N. B. Wagner M. (2019). When fluorescence is not a particle: the tissue translocation of microplastics in *Daphnia magna* seems an artifact. Environ. Toxicol. Chem. 38, 1495–1503. 10.1002/etc.4436 31009098

[B110] Schwabl P. Köppel S. Königshofer P. Bucsics T. Trauner M. Reiberger T.. (2019). Detection of various MPs in human stool: a prospective case series. Ann. Intern. Med. 171, 453–457. 10.7326/M19-0618 31476765

[B111] Shim W. J. Song Y. K. Hong S. H. Jang M. (2016). Identification and quantification of MPs using Nile red staining. Mar. Pollut. Bull. 113, 469–476. 10.1016/j.marpolbul.2016.10.049 28340965

[B112] Shruti V. C. Jonathan M. P. Rodriguez-Espinosa P. F. Rodríguez-González F. (2019). MPs in freshwater sediments of Atoyac River basin, Puebla City, Mexico. Sci. Total Environ. 654, 154–163. 10.1016/j.scitotenv.2018.11.054 30445318

[B113] Sims P. A. Hardin J. D. (2007). Fluorescence-integrated transmission electron microscopy images: integrating fluorescence microscopy with transmission electron microscopy. Methods Mol. Biol. (Clifton, N.J.), 369, 291–308. 10.1007/978-1-59745-294-6_14 17656756

[B114] Smith B. C. (2011). Fundamentals of Fourier Transform Infrared Spectroscopy. Boca Raton, FL: CRC Press. 10.1201/b10777

[B115] Song C. Liu Z. Wang C. Li S. Kitamura Y. (2020). Different interaction performance between MPs and microalgae: the bio-elimination potential of Chlorella sp. L38 and *Phaeodactylum tricornutum* MASCC-0025. Sci. Total. Environ. 723:138146. 10.1016/j.scitotenv.2020.138146 32222515

[B116] Song Y. K. Hong S. H. Jang M. Han G. M. Rani M. Lee J.. (2015). A comparison of microscopic and spectroscopic identification methods for analysis of MPs in environmental samples. Mar. Pollut. Bull. 93, 202–209. 10.1016/j.marpolbul.2015.01.015 25682567

[B117] Song Y. K. Hong S. H. Jang M. Kang J. H. Kwon O. Y. Han G. M.. (2014). Large accumulation of micro-sized synthetic polymer particles in the sea surface microlayer. Environ. Sci. Technol. 48, 9014–9021. 10.1021/es501757s 25059595

[B118] Stock V. Böhmert L. Lisicki E. Block R. Carmona J. C. Pack L. K.. (2019). Uptake and effects of orally ingested polystyrene microplastic particles *in vitro* and *in vivo*. Arch. Toxicol. 93, 1817–1833. 10.1007/s00204-019-02478-7 31139862

[B119] Sun X. Chen B. Li Q. Liu N. Xia B. Zhu L.. (2018). Toxicities of polystyrene nano- and MPs toward marine bacterium *Halomonas alkaliphila*. Sci. Total. Environ. 642, 1378–1385. 10.1016/j.scitotenv.2018.06.141 30045518

[B120] Syberg K. Khan F. R. Selck H. Palmqvist A. Banta G. T. Daley J.. (2015). MPs: addressing ecological risk through lessons learned. Environ. Toxicol. Chem. 34, 945–953. 10.1002/etc.2914 25655822

[B121] Tenuta T. Monopoli M. P. Kim J. Salvati A. Dawson K. A. Sandin P.. (2011). Elution of labile fluorescent dye from nanoparticles during biological use. PLoS ONE 6:e25556. 10.1371/journal.pone.0025556 21998668 PMC3188558

[B122] Tiwari M. Rathod T. D. Ajmal P. Y. Bhangare R. C. Sahu S. K. (2019). Distribution and characterisation of MPs in beach sand from three different Indian coastal environments. Mar. Pollut. Bull. 140, 62–273. 10.1016/j.marpolbul.2019.01.055 30803642

[B123] Tong H. Jiang Q. Hu X. Zhong X. (2020). Occurrence and identification of MPs in tap water from China. Chemosphere 252:126493. 10.1016/j.chemosphere.2020.126493 32199168

[B124] Trotsenko O. Koestner R. Roiter Y. Tokarev A. Minko S. (2016). Probing rough composite surfaces with atomic force microscopy: Nafion ionomer in fuel cell electrodes. Polymer 102, 396–403. 10.1016/j.polymer.2015.11.021

[B125] Turner A. Holmes L. (2011). Occurrence, distribution and characteristics of beached plastic production pellets on the island of Malta (central Mediterranean). Mar. Pollut. Bull. 62, 377–381. 10.1016/j.marpolbul.2010.09.027 21030052

[B126] Ukraintsev E. Kromka A. Kozak H. Remeš Z. Rezek B. (2012). Artifacts in atomic force microscopy of biological samples. Atomic force microscopy investigations into biology—from cell to protein. Intech Rijeka 29–54. 10.5772/36203

[B127] Van Cauwenberghe L. Vanreusel A. Mees J. Janssen C. R. (2013). Microplastic pollution in deep-sea sediments. Environ. Pollut. 182, 495–499. 10.1016/j.envpol.2013.08.013 24035457

[B128] Veneman W. J. Spaink H. P. Brun N. R. Boskera T. Vijver M. G. (2017). Pathway analysis of systemic transcriptome responses to injected polystyrene particles in zebrafish larvae. Aquat. Toxicol. 190, 112–120. 10.1016/j.aquatox.2017.06.014 28704660

[B129] Wagner J. Wang Z. M. Ghosal S. Rochman C. Gassel M. Wall S. (2017). Novel method for the extraction and identification of microplastics in ocean trawl and fish gut matrices. Anal. Methods. 9, 1479–1490. 10.1039/C6AY02396G28646780

[B130] Wang J. Coffin S. Sun C. Schlenk D. Gan J. (2019). Negligible effects of microplastics on animal fitness and HOC bioaccumulation in earthworm Eisenia fetida in soil. Environ. Pollu. 249, 776–784. 10.1016/j.envpol.2019.03.102 30951961

[B131] Wang W. Wang J. (2018). Investigation of MPs in aquatic environments: an overview of the methods used, from field sampling to laboratory analysis. Trends Anal. Chem. 108, 195–202. 10.1016/j.trac.2018.08.026

[B132] Wu B. Wu X. Liu S. Wang Z. Chen L. (2019). Size-dependent effects of polystyrene MPs on cytotoxicity and efflux pump inhibition in human Caco-2 cells. Chemosphere 221, 333–341. 10.1016/j.chemosphere.2019.01.056 30641374

[B133] Wu P. Huang J. Zheng Y. Yang Y. Zhang Y. He F.. (2019). Environmental occurrences, fate, and impacts of MPs. Ecotoxicol. Environ. Saf. 184:109612. 10.1016/j.ecoenv.2019.109612 31476450

[B134] Yang L. Zhang Y. Kang S. Wang Z. Wu C. (2020). MPs in freshwater sediment: a review on methods, occurrence, and sources. Sci. Total Environ. 754:141948. 10.1016/j.scitotenv.2020.141948 32916488

[B135] Yang R. Wang B. Li M. D. Zhang X. Li J. C. (2019). Preparation, characterization and thermal degradation behavior of rigid polyurethane foam using a malic acid based polyols. Ind. Crops Prod. 136, 121–128. 10.1016/j.indcrop.2019.04.073

[B136] Yin L. Wen X. Du C. Jiang J. Wu L. Zhang Y.. (2020). Comparison of the abundance of MPs between rural and urban areas: a case study from East Dongting Lake. Chemosphere 244:125486. 10.1016/j.chemosphere.2019.125486 31812050

[B137] Yong C. Q. Y. Valiyaveetill S. Tang B. L. (2020). Toxicity of MPs and nanoplastics in mammalian systems. Int. J. Environ. Res. Public Health 17:1509. 10.3390/ijerph17051509 PMC7084551 32111046

[B138] Zhang D. Liu X. Huang W. Li J. Wang C. Zhang D.. (2020). Microplastic pollution in deep-sea sediments and organisms of the Western Pacific Ocean. Environ. Pollut. 259:113948. 10.1016/j.envpol.2020.113948 32023798

[B139] Zhang W Crittenden J. Li K. Chen Y. (2012). Attachment efficiency of nanoparticle aggregation in aqueous dispersions: modeling and experimental validation. Environ. Sci. Technol. 46, 7054–7062. 10.1021/es203623z 22260181

[B140] Ziajahromi S. Kumar A. Neale P. A. Leusch F. D. L. (2018). Environmentally relevant concentrations of polyethylene MPs negatively impact the survival, growth and emergence of sediment-dwelling invertebrates. Environ. Pollut. 236, 425–431. 10.1016/j.envpol.2018.01.094 29414367

